# Development and characterization of microsatellite loci for the haploid–diploid red seaweed *Gracilaria vermiculophylla*

**DOI:** 10.7717/peerj.1159

**Published:** 2015-08-11

**Authors:** Nicole M. Kollars, Stacy A. Krueger-Hadfield, James E. Byers, Thomas W. Greig, Allan E. Strand, Florian Weinberger, Erik E. Sotka

**Affiliations:** 1 Grice Marine Laboratory and the Department of Biology, College of Charleston, Charleston, SC, USA; 2 Odum School of Ecology, University of Georgia, Athens, GA, USA; 3 Center for Coastal Environmental Health and Biomolecular Research, National Oceanic and Atmospheric Administration, Charleston, SC, USA; 4 Helmholtz-Zentrum fur Ozeanforschung Kiel (GEOMAR), Kiel, Germany; 5 Current affiliation: Center for Population Biology, University of California, Davis, CA, USA

**Keywords:** Complex life cycles, Biological invasions, Seaweed, Microsatellites, Haploid-diploid, *Gracilaria vermiculophylla*

## Abstract

Microsatellite loci are popular molecular markers due to their resolution in distinguishing individual genotypes. However, they have rarely been used to explore the population dynamics in species with biphasic life cycles in which both haploid and diploid stages develop into independent, functional organisms. We developed microsatellite loci for the haploid–diploid red seaweed *Gracilaria vermiculophylla*, a widespread non-native species in coastal estuaries of the Northern hemisphere. Forty-two loci were screened for amplification and polymorphism. Nine of these loci were polymorphic across four populations of the extant range with two to eleven alleles observed. Mean observed and expected heterozygosities ranged from 0.265 to 0.527 and 0.317 to 0.387, respectively. Overall, these markers will aid in the study of the invasive history of this seaweed and further studies on the population dynamics of this important haploid–diploid primary producer.

## Introduction

In the last decade, genetic approaches to answering evolutionary and ecological questions have become less expensive and more easily applied to non-model species (Allendorf, Hohenlohe & Luikart, 2010; Guichoux et al., 2011). Microsatellites, or tandem repeats of two to six nucleotides, are popular molecular markers due to their resolution in distinguishing individual genotypes (Selkoe & Toonen, 2006) and their ability to describe patterns of population connectivity across landscapes (Manel et al., 2003) and seascapes (Galindo, Olson & Palumbi, 2006). Much of the literature focuses on organisms with single free-living diploid stages (i.e., animals and higher plants). Yet, there are many species with both haploid and diploid stages in the same life cycle in which both ploidies undergo somatic development and live as independent, functional organisms.

While theory predicts that selection should favor either diploidy or haploidy (Mable & Otto, 1998), Hughes & Otto (1999) demonstrated the maintenance of both haploid and diploid stages when the two stages occupy different ecological niches. However, there are relatively few empirical tests of these alternative hypotheses (but see Destombe et al., 1992; Thornber & Gaines, 2004; Guillemin et al., 2013), and for isomorphic species in which ploidy is not easily identified through morphological traits, molecular markers will be essential to advance research in this field. These same markers can additionally be used to understand connectivity and demographic history in haploid–diploid populations. Among marine haploid–diploid macroalgae, relatively few microsatellites have been developed to address any of these issues (but see Table 1).

**Table 1:** Studies in which both the haploid and diploid stages of seaweeds and mosses were investigated to reveal patterns in genetic structure and mating system.

	Phylum	Species	Marker	Type of study
Sosa et al. (1998)	Rhodophyta	*Gelidium arbuscula*	Isozymes	Genetic structure and mating system
Sosa et al. (1998)	Rhodophyta	*Gelidium canariensis*	Isozymes	Genetic structure and mating system
Engel et al. (1999)	Rhodophyta	*Gracilaria gracilis*	Microsatellites	Paternity analyses and dispersal
van der Velde et al. (2001)	Bryophyta	*Polytrichum formosum*	Microsatellites	Paternity analyses and dispersal
van der Strate et al. (2002)	Chlorophyta	*Cladophoropsis membranacea*	Microsatellites	Shorescape structure and mating system
Engel, Destombe & Valero (2004)	Rhodophyta	*Gracilaria gracilis*	Microsatellites	Shorescape structure and mating system
Guillemin et al. (2008a) and Guillemin et al. (2008b)	Rhodophyta	*Gracilaria chilensis*	Microsatellites	Genetic structure, mating system and comparisons between natural and farmed populations
Szövényi, Ricca & Shaw (2009)	Bryophyta	*Sphagnum lescurii*	Microsatellites	Paternity analyses and dispersal
Alström-Rapaport, Leskinen & Pamilo (2010)	Chlorophyta	*Ulva intenstinalis*	Microsatellites	Genetic structure and mating system
Krueger-Hadfield et al. (2011)	Rhodophyta	*Chondrus crispus*	Microsatellites	Genetic structure and mating system
Krueger-Hadfield et al. (2013)	Rhodophyta	*Chondrus crispus*	Microsatellites	Shorescape structure and mating system
Couceiro et al. (2015)	Ochrophyta	*Ecotcarpus crouaniorum*	Microsatellites	Genetic structure and mating system
Couceiro et al. (2015)	Ochrophyta	*Ectocarpus siliculosus*	Microsatellites	Genetic structure and mating system
Krueger-Hadfield et al. (2015)	Rhodophyta	*Chondrus crispus*	Microsatellites	Paternity analyses and dispersal

Understanding the consequences of biphasic life cycles and land- or seascape features on population structure is particularly relevant in light of the increasing frequency of biological introductions. There are numerous examples of widespread, and putatively invasive species, that have free-living haploid and diploid stages, including macroalgae (e.g., *Asparagopsis* spp.; Andreakis et al., 2007), ferns (e.g., *Lygodium* spp.; Lott et al., 2003) and mosses (e.g., *Campylopus introflexus*; Schirmel, Timler & Buchholz, 2010). Macroalgae, or seaweeds, account for approximately 20% of the world’s introduced marine species (Andreakis & Schaffelke, 2012) and a subset of these invasions are by species that are exploited in their native range, either for the phycocolloid industry or as food products (Williams & Smith, 2007).

The red seaweed *Gracilaria vermiculophylla* (Omhi) Papenfuss is native to the northwest Pacific and, in the last 30–40 years, has spread throughout high to medium salinity estuaries of the eastern North Pacific (Saunders, 2009), the western North Atlantic (Byers et al., 2012) and the eastern North Atlantic (Weinberger et al., 2008; Guillemin et al., 2008a). *G. vermiculophylla* transforms the ecosystems into which it is introduced through negative impacts on native species (e.g., direct competition, Hammann et al., 2013), the addition of structural complexity to soft-bottom systems (e.g., Nyberg, Thomsen & Wallentinus, 2009; Wright et al., 2014) and the alteration of community structure, species interactions and detrital pathways (e.g., Byers et al., 2012). Previous studies of the population genetics of *G. vermiculophylla* focused on the mitochondrial gene *cytochrome b oxidase I* (Kim, Weinberger & Boo, 2010; Gulbransen et al., 2012), but mitochondrial genetics do not necessarily predict the population genetics of the nuclear genome and cannot assess patterns of ploidy and mating system. Thus, we developed nine polymorphic microsatellite loci for *G. vermiculophylla*.

**Table 2:** Characteristics of nine polymorphic microsatellite loci developed for *Gracilaria vermiculophylla*. Acc. No., GenBank accession number; locus; motif; primer sequences; allele range; avg. error: TANDEM (Matschiner & Saltzburger, 2009) rounding errors for each microsatellite locus (the authors of TANDEM suggest that good loci have an average rounding error which is below 10% of the repeat size); *N*_tall_, total number of alleles. All loci showed one-locus genetic determinism.

Locus	Acc. No.	Motif	Primer sequence	Allele range	Avg. Error	*N*_**tall**_
Gverm_5276	KT232089	(AC)_10_	F: GGAGAGCAGCACGTTTTAGG R: CTGCTTAGTTCCACGATCGAC	282–316	0.14	11
Gverm_6311	KT232090	(AG)_9_	F: GCGTCATTCCACTGAATGTG R: GATGAACCTCAATGCCTCGT	203–223	0.17	6
Gverm_8036	KT232091	(AC)_12_	F: GCCCTTTTAAGGATGCAACA R: GGGGTAAACGACCACAGAGA	213–251	0.14	5
Gverm_3003	KT232092	(AG)_11_	F: CATCTTGCTTCCTTGCTTCC R: TTGAAAGCCGGAATTTATCG	198–230	0.11	4
Gverm_1203	KT232093	(AAG)_8_	F: CTCCTGGTGCACAAGCAATA R: ACATTCTGCGCACCTTTCTT	284–308	0.12	4
Gverm_1803	KT232094	(AC)_11_	F: GCGTGCACGATGTCTACACT R: GACAGCAACAAGTGGGGTTT	352–356	0.07	3
Gverm_804	KT232095	(AAG)_8_	F: TGTAGGATTGCTCTCCTGGTG R: CAGGCTGGCCAAAATAACAT	182–188	0.16	3
Gverm_10367	KT232096	(AG)_8_	F: GCTGAGAAATGAAGCGAAGG R: GCAAACCTGCCTTGTTTGTT	198–200	0.07	2
Gverm_2790	KT232097	(AATGC)_5_	F: GAACAATGCGGGAAAACATT R: GGAAGAGGCTCAAAAGCAGA	262–267	0.16	2

## Materials and Methods

A library of contigs for *G. vermiculophylla* was generated using the 454 next-generation sequencing platform (Cornell University Life Sciences Core Laboratory Center) from a single individual collected from Charleston, South Carolina, USA. For library preparation, DNA was extracted using CTAB (Eichenberger, Gugerli & Schneller, 2000) and library construction followed Hamilton et al. (1999). Dimeric to hexameric microsatellite repeats were identified with the program MSATCOMMANDER, ver 1.0.8 (Faircloth, 2008) and primers were designed using PRIMER 3 (Rozen & Skalesty, 2000) for contigs with at least four sequences present in the library. Bioinformatics of these sequences was facilitated by the APE package (Paradis, Claude & Strimmer, 2004) in *R* (R Development Core Team, 2014).

Total genomic DNA was isolated using 120 µL of a 10% Chelex solution (BioRad Laboratories, Hercules, California, USA) in which approximately 1 cm of dried algal tissue was heated at 95 °C for 30 min and vortexed intermittently (Walsh, Metzger & Higuchi, 1991). Loci were amplified on a thermocycler (BioRad) as follows: 10 µL final volume, 2 µL of stock DNA template, 0.5 units of GoTAQ Flexi-DNA Polymerase (Promega, Madison, Wisconsin, USA), 1X buffer, 250 µM of each dNTP, 1.5 nM of MgCl_2_, 150 nM of fluorescently-labeled forward primer, 100 nM of unlabeled forward primer and 250 nM of unlabeled reverse primer. The PCR program included 2 min at 95 °C, 30 cycles of 30 s at 95 °C, 30 s at 55 °C and 30 s at 72 °C, and a final 5 min at 72 °C. One µL of each PCR product was added to 10 µL of loading buffer containing 0.35 µL of size standard (GeneScan500 Liz; Applied Biosystems, Foster City, California, USA). Samples were electrophoresed on an ABI 3130xL genetic analyzer equipped with 36 cm capillaries (Applied Biosystems). Alleles were scored manually using GENEMAPPER ver. 4 (Applied Biosystems) and allele sizes were binned with TANDEM ver. 1.08 software (Matschiner & Saltzburger, 2009; Krueger-Hadfield et al., 2013).

We screened a total of 42 primer pairs for amplification and polymorphism in *G. vermiculophylla* (Table 2, Table S1). For the amplifiable loci that also showed polymorphism (nine total, see “Results and Discussion”), we verified single locus genetic determinism (SGLD). Loci were in SLGD if known haploids produced a single allele and diploids produced either one or two alleles in their homozygous or heterozygous state, respectively. We verified SGLD in a subset of known haploid gametophytes (*n* = 28) and diploid tetrasporophytes (*n* = 30) collected at Elkhorn Slough, California, USA (Table 3, Fig. S1). Elkhorn Slough was the only population for which ploidy was determined by reproductive structures and for which we had known haploids and diploids for genotyping.

**Table 3:** Location of the four populations used to test for polymorphism in newly characterized microsatellite loci in *Gracilaria vermiculophylla*. The region, range (native or non-native), latitude, longitude, sampling date, collector* and ploidy determination (using reproductive phenology or microsatellite genotype) are provided.

Population	Region	Range	Latitude	Longitude	Date	Collector	Ploidy determination
Akkeshi, Japan	NW Pacific	Native	43.04774	144.9498	25 Aug 10, 31 Jul 12	NMK, KH, KM, AP, MS	Genotype
Elkhorn Slough California, USA	NE Pacific	Non-native	36.50447	−121.4513	3 Nov 13	SAKH, BFK, TDK, BH	Genotype, phenology
Fort Johnson SC, USA	NW Atlantic	Non-native	32.7513	−79.900	11 Dec 13	CEG	Genotype
Nordstrand, Germany	North Sea	Non-native	54.454571	8.874846	24 Mar 10	MH	Genotype

**Notes.**

Collector abbreviations NMK NM Kollars KH K Honda KM K Momota AP A Pansch MS M Sato SAKH SA Krueger-Hadfield BFK BF Krueger TDK TD Krueger BH B Hughes CEG CE Gerstenmaier MH: M Hammann

The frequency of null alleles was estimated in the haploid subpopulation from Elkhorn Slough as well as diploid tetrasporophytes for each of the four populations (Table 3). It is possible to calculate the null allele frequency directly in the haploids based on the number of non-amplification events, after discounting technical errors. For diploid tetrasporophytes, we used a maximum likelihood estimator (ML-NullFreq: Kalinowski & Taper, 2006).

Next, we screened loci for short allele dominance (Wattier et al., 1998). The presence of short allele dominance is rarely tested during microsatellite development, even though it can result in artificial heterozygote deficiencies. In contrast to null alleles, primer binding is successful, but the larger allele is not amplified due to the preferential amplification of the smaller allele. Wattier et al. (1998) demonstrated an analytical method to detect short allele dominance using linear models. If a regression of allele-specific *F_is_* (inbreeding coefficient) statistics on allele size reveals a significant negative slope, then short allele dominance may be expected. We determined three to four allele size classes per locus and performed linear regressions using the STATS package in *R* (R Development Core Team, 2014).

To provide preliminary assessment of the genotypic and genetic diversity one can gain from these loci, we genotyped diploid tetrasporophytes from one native and three non-native populations of *G. vermiculophylla* (Table 3). Diploids were identified based either on reproductive phenology (Elkhorn) or microsatellite genotype (after assuring SGLD) if at least one locus was heterozygous (Akkeshi, Fort Johnson and Nordstrand, Table 3).

We calculated expected allelic richness using rarefaction in order to account for differences in sample size (HP-Rare; Kalinowski, 2005). Observed (*H_O_*) and expected heterozygosities (*H_E_*) were calculated using GenAlEx, ver. 6.501 (Peakall & Smouse, 2006; Peakall & Smouse, 2012). Tests for Hardy–Weinberg equilibrium and *F*-statistics were performed in FSTAT, ver. 2.9.3.2 (Goudet, 1995). *F_is_* was calculated for each locus and over all loci according to Weir & Cockerham (1984) and significance (at the adjusted nominal level of 0.001) was tested by running 1,000 permutations of alleles among individuals within samples. We also tested for linkage disequilibrium in each population using GENEPOP, ver. 4.2.2 (Rousset, 2008), with 1,000 permutations followed by Bonferroni correction for multiple comparisons (Sokal & Rohlf, 1995).

## Results and Discussion

Of the 42 loci screened, 16 did not amplify for *G. vermiculophylla* even after several PCR modifications (Table S1). Of the remaining 26 loci, four loci exhibited multi-peak profiles and were discarded from further use, 13 loci were considered monomorphic (Table S1), and nine loci showed polymorphism (Table 2). The nine polymorphic loci exhibited SLGD in which known haploids always exhibited one allele. The low number of polymorphic loci revealed from this screening process is consistent with previous efforts to develop microsatellite loci for some seaweeds (e.g., Varlea-Álvarez et al., 2011; Arnaud-Haond et al., 2013).

The frequency of null alleles was zero at all loci except Gverm_1803 and Gverm_2790 in which the frequencies were both 0.019 in the haploids at Elkhorn Slough (Table S2). The only evidence of null alleles in the diploids from Elkhorn Slough was at locus Gverm_1803, with a maximum likelihood estimated frequency of 0.115. The discrepancy between the haploid and diploid estimates is likely due to assumptions underlying the maximum likelihood estimators implemented in software like HP-Rare (Kalinowski, 2005), such as random mating. Krueger-Hadfield et al. (2013) demonstrated a strong bias in the estimates of null allele frequency when using these maximum likelihood estimators in macroalgal populations that have undergone non-random mating. The higher frequencies of null alleles (0.115–0.207) in the Akkeshi diploid subpopulation were most likely driven by a violation of these assumptions as well, though empirical estimates in haploid subpopulations are warranted. Nevertheless, the low frequency of null alleles and lack of evidence for short-allele dominance (all regression *p*-values were >0.2, Table S3), suggest that observed heterozygote deficiencies using these loci will be due to the mating system or spatial substructuring (Guillemin et al., 2008b; Krueger-Hadfield et al., 2011; Krueger-Hadfield et al., 2013).

Previous studies have used microsatellite loci to distinguish among individual clones and to describe the genetic diversity and the mating systems of seaweed populations despite low levels of polymorphism (e.g., Guillemin et al., 2008b; Arnaud-Haond et al., 2013). In the current study, the nine polymorphic markers described genetic variability in four populations sampled across the extant distribution of *G. vermiculophylla*. Overall, there was little evidence for linkage disequilibrium after Bonferroni correction (Table S4). Additionally, allelic diversity was comparable among the one native and three non-native sites we sampled, but *F_is_* varied considerably (summary in Table 4; per locus statistics in Table S5). Together, these results suggest that unique demographic and evolutionary processes could be operating between native and non-native ranges and within each population, but more detailed sampling is needed to address these patterns.

**Table 4:** Genetic features of four populations of *Gracilaria vermicuolphylla*. These include: the sample size, *N*; the diploid genotypic richness, *N_A_*, + standard error (SE); mean allelic richness, *A_E_*, based on the smallest sample size of 46 alleles (23 diploid individuals) + SE; mean observed heterozygosity, *H_O_*, + SE; mean expected heterozygosity, *H_E_*, + SE; inbreeding oefficient, *F_is_*, multilocus and per locus estimates.

Statistics	Akkeshi	Elkhorn slough	Fort Johnson	Nordstrand
*N*	31	30	38	23
*N_A_*	3.2 ± 0.5	2.2 ± 0.4	2.0 ± 0.2	1.9 ± 0.2
*A_E_*	3.1 ± 0.4	2.2 ± 0.3	2.0 ± 0.2	1.9 ± 0.2
*H_O_*	0.265 ± 0.060	0.311 ± 0.089	0.520 ± 0.110	0.527 ± 0.125
*H_E_*	0.374 ± 0.079	0.317 ± 0.084	0.387 ± 0.077	0.352 ± 0.079
*F_is_*	0.294 ^*^	0.017	−0.350 ^*^	−0.512 ^*^
***F***_***is***_ **per locus**				
Gverm_5276	0.484 ^*^	0.120	−0.209	−0.492
Gverm_6311	0.435 ^*^	0.140	−0.267	−0.048
Gverm_8036	0.334	NA	−0.445 ^*^	−0.217
Gverm_3003	0.529	−0.121	−0.138	−0.553
Gverm_1203	−0.15	−0.206	−0.310	−0.508
Gverm_1803	0.569 ^*^	0.460	−0.696 ^*^	NA
Gverm_804	−0.278	−0.206	−0.310	−0.508
Gverm_10367	−0.017	NA	NA	NA
Gverm_2790	NA	NA	NA	−0.913^*^

**Notes.**

* *p* < 0.001, adjusted nominal value.

In summary, we have developed and characterized microsatellite markers for the haploid–diploid red seaweed *G. vermiculophylla*. These loci have the resolution to distinguish individual thalli and will aid studies on the invasive history of *G. vermiculophylla*, as well as the evolutionary ecology of rapidly spreading populations and mating system shifts in organisms that have biphasic life cycles with free-living haploid and diploid stages (i.e., macroalgae, ferns, mosses and some fungi).

## Supplemental Information

10.7717/peerj.1159/supp-1 Figure S1 Reproductive phenology of (A) haploid female gametophytes, (B) haploid male gametophytes (50x) and (C) diploid tetrasporophytes (50x) *Gracilaria vermiculophylla* exhibits a complex haploid–diploid life cycle, including two free-living stages of different ploidy levels: the haploid dioecious gametophytes and the diploid tetrasporophytes. Fertilization takes place on the female gametophyte, and the female gametes, once fertilized, develop into cystocarps, an additional, though not a free-living, stage. The cystocarp is a macroscopic swelling on the surface of the female thallus, within which the zygote is mitotically copied producing thousands of diploid spores, called carpospores. Spores released from the cystocarps germinate into the diploid tetrasporophytes. (Photo credit: SA Krueger-Hadfield).

10.7717/peerj.1159/supp-2 Table S1 Characteristics of 33 microsatellite loci developed for *Gracilaria vermiculophylla* that showed monomorphism, non-amplification, or multi-locus genetic determinism Acc. No., GenBank accession number; locus; motif; primer sequences; Profile: one- or multi-locus genetic determinism, no amp. indicates non-amplification; *N*_tall_ total number of alleles.

10.7717/peerj.1159/supp-3 Table S2 Null allele frequencies for the microsatellite loci developed for *Gracilaria vermiculophylla* Frequencies were directly estimated in the haploid subpopulations, whereas frequencies in each of the diploid subpopulations at Akkeshi, Elkhorn Slough, Fort Johnson and Nordstrand were calculated using maximum likelihood and the software MLNullFreq (Kalinowski & Taper, 2006).

10.7717/peerj.1159/supp-4 Table S3 Short allele dominance analysis for microsatellite loci developed for *Gracilaria vermiculophylla* This includes number of pooled size classes used in regression analysis (following Wattier et al., 1998), *No. of classes*, and linear regression statistics. Loci Gverm_10367 and Gverm_2790 only exhibited two alleles in our sampled populations and consequently, short allele dominance analysis was not applicable (NA).

10.7717/peerj.1159/supp-5 Table S4 Linkage disequilibrium analysis for microsatellite loci developed for *Gracilaria vermiculophylla* Darkened cells indicate pairs of loci that show significant linkage disequilibrium after Bonferroni correction (*p*-value threshold < 0.006 at *α* = 0.05).

10.7717/peerj.1159/supp-6 Table S5 Genetic features per locus of four populations of *Gracilaria vermiculophylla* This includes: number of alleles at each locus, *N_A_*, + standard error (SE); mean allelic richness, *A_E_*, based on the smallest global sample size of 46 alleles (23 diploid individuals) + SE; mean observed heterozygosity, *H_O_*, + SE; mean expected heterozygosity, *H_E_*, + SE.

10.7717/peerj.1159/supp-7 Supplemental Information 1 Development and characterization of microsatellite loci for the haploid–diploid red seaweed *Gracilaria vermiculophylla*

## Additional Information and Declarations

### DNA Deposition

The following information was supplied regarding the deposition of DNA sequences: GenBank accession numbers can be found in Table 2.
