# Supplementary figures and images for: Development and characterization of microsatellite loci for the haploid–diploid red seaweed Gracilaria vermiculophylla
Source: PeerJ. 2015 Aug 11;3:e1159. doi: 10.7717/peerj.1159 (PMC4558075; doi:10.7717/peerj.1159)

A)

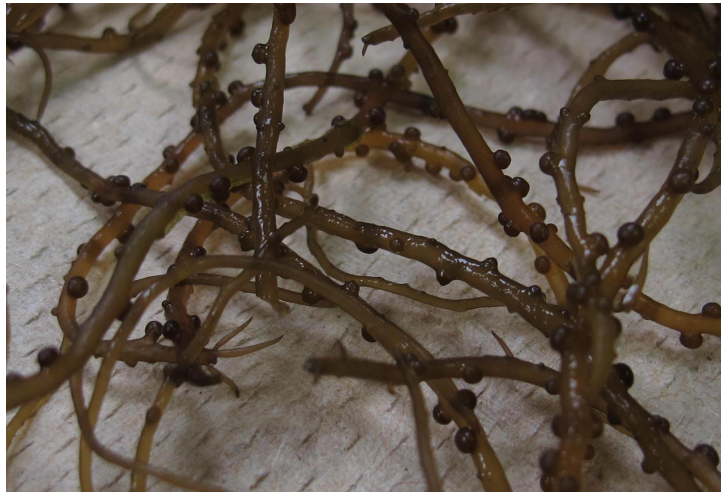

B)

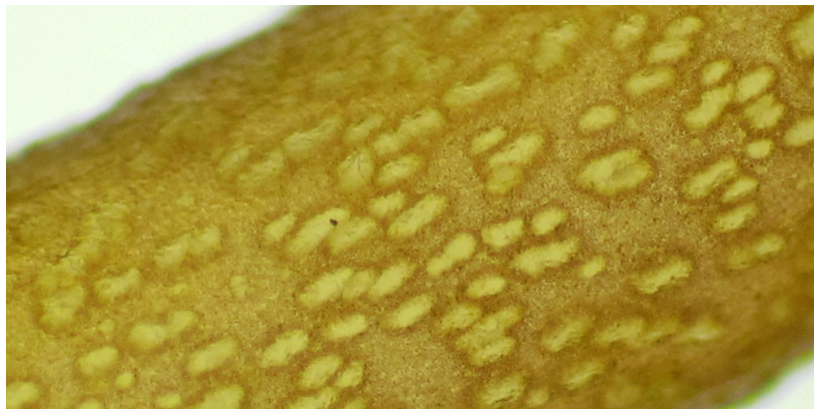

C)

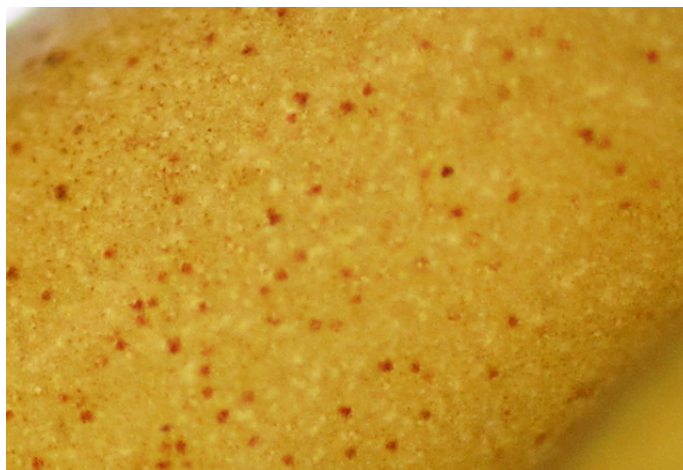

Supplement: Figure S1 — Gracilaria vermiculophylla exhibits a complex haploid–diploid life cycle, including two free-living stages of different ploidy levels: the haploid dioecious gametophytes and the diploid tetrasporophytes. Fertilization takes place on the female gametophyte, and the female gametes, once fertilized, develop into cystocarps, an additional, though not a free-living, stage. The cystocarp is a macroscopic swelling on the surface of the female thallus, within which the zygote is mitotically copied producing thousands of diploid spores, called carpospores. Spores released from the cystocarps germinate into the diploid tetrasporophytes. (Photo credit: SA Krueger-Hadfield). [file peerj-03-1159-s001.pdf]
