# Supplementary material for: Development and characterization of microsatellite loci for the haploid–diploid red seaweed Gracilaria vermiculophylla
Source: PeerJ. 2015 Aug 11;3:e1159. doi: 10.7717/peerj.1159 (PMC4558075; doi:10.7717/peerj.1159)
Supplement: Table S1 — Acc. No., GenBank accession number; locus; motif; primer sequences; Profile: one- or multi-locus genetic determinism, no amp. indicates non-amplification; Ntall total number of alleles. [file peerj-03-1159-s002.docx]

Table S1. Characteristics of 33 microsatellite loci developed for *Gracilaria vermiculophylla* that showed monomorphism, non-amplification, or multi-locus genetic determinism: Acc. No. = genbank accession number; locus; motif; primer sequences; Profile: one- or multi-locus genetic determinism, no amp. indicates non-amplification; *N­tall* = total number of alleles.

| **Locus** | **Acc. No.** | **Motif** | **Primer sequence** | **Profile** |
| --- | --- | --- | --- | --- |
| Gverm_10883 | KT232098 | (AAG)_7_ | F: TCCCATCAGCCAACAGTAGA  R: GCAGAGCTTGGTAGGCATTC | one |
| Gverm_7969 | KT232099 | (ATC)_6_ | F: CGATCCTTCCTCCTGTGGTA  R: AATTGGGATACGCAATACGG | one |
| Gverm_13240 | KT232100 | (ACG)_8_ | F: AACACATTGCTTCCGTTCTTG  R: CTGCGAAGCACAAGTGATGT | one |
| Gverm_2178 | KT232101 | (ACG)_6_ | F: ATTTGCACCGGTAAAACTGG  R: GGCTGTCATGCAAGATGATG | one |
| Gverm_12220 | KT232102 | (AC)_7_ | F: TGACTCGAGGAGTGCAGATG  R: CTTTTGCCAGCAATGCAATA | one |
| Gverm_10926 | KT232103 | (AC)_7_ | F: CTTTTGCCAGCAATGCAATA  R: TGACTCGAGGAGTGCAGATG | one |
| Gverm_10612 | KT232104 | (AAG)_7_ | F: GCAGAGCTTGGTAGGCATTC  R: GGCAACACCATTGGACTCTT | one |
| Gverm_12990 | KT232105 | (AC)_7_ | F: GGGCGTAGAGAGCTGAAAGA  R: TCGCCGTTTTTCTCCTACAC | one |
| Gverm_9808 | KT232106 | (AG)_10_ | F: GCCTGTTCCTCATCTTTTGG  R: GCGACAGAAGAGGCGACTTA | one |
| Gverm_10115 | KT232107 | (AG)_8_ | F: CAGGGGCTACTCACCTTCAC  R: GTGTGTCTTGATCCGCTGTG | one |
| Gverm_10134 | KT232108 | (AC)_8_ | F: CCGAAAGATTAGCGATCCAC  R: CTCCCCCTCTTGGTTTTGTT | one |
| Gverm_871 | KT232109 | (AG)_9_ | F: ACACGGGTCTCATGTTCCTC  R: AGGAGCGCAGTCCAAGTAAA | one |
| Gverm_3707 | KT232110 | (AG)_10_ | F: ACGACTCACGGGTTGTTTTT  R: AAGCAGACCAGCACATTTCA | one |
| Gverm_4346 | KT232111 | (AC)_8_ | F: ATCGCTCTTCTTCGGCTACA  R: AGGGATGACTCACCAAGTGC | multi |
| Gverm_263 | KT232112 | (ACG)_9_ | F: CACATTGCTTCCGTTCTTGTT  R: TGAGTTCGTCGTCACGATTC | multi |
| Gverm_12453 | KT232113 | (AG)_13_ | F: GAAGACTGACCGGAATCTGC  R: ACAATCAACACGCAGCTCAG | multi |
| Gverm_13408 | KT232114 | (AAG)_9_ | F: GGGAACGCATATCTTTGTGG  R: GATACGGGAACAGCGTTTGT | multi |
| Gverm_8854 | KT232115 | (AAAT)_5_ | F: TGCGCAGGAAAGGGTTAATA  R: ATCCCTCGTGATAGGCAAAT | no amp. |
| Gverm_6659 | KT232116 | (AC)_6_ | F: TGGTACGTGATCCCAGTGTG  R: CATCGTGCTTCACCACATTC | no amp. |
| Gverm_85 | KT232117 | (ACC)_6_ | F: AAAGTGGAGGCAGCTATGGA  R: TCCCCAATGAACTCATGACA | no amp. |
| Gverm_5509 | KT232118 | (AC)_9_ | F: ACTTTGCACCTTTGCACCTT  R: GTTGGAATGGTTCTGCGATT | no amp. |
| Gverm_269 | KT232119 | (ACG)_9_ | F: CACATTGCTTCCGTTCTTGTT  R: TGAGTTCGTCGTCACGATTC | no amp. |
| Gverm_3258 | KT232120 | (AC)_9_ | F: AACTGGAGCTTGGGATGCTA  R: CCGTCTCTGTTTCTTTCTAGTGC | no amp. |
| Gverm_3883 | KT232121 | (ACAG)_20_ | F: ATCGAGATATTTACACGCAACA  R: AGAGGGCAGTTAGATTGTCTGA | no amp. |
| Gverm_5516 | KT232122 | (AAC)_8_ | F: AGTTGTAACAGCGGGAAACG  R: CCCTGTTGCGAATCTTCTGT | no amp. |
| Gverm_6564 | KT232123 | (AAC)_13_ | F: CCCTAAGCTCTGCCATTGTC  R: TGCGCTGTAACAAGAAGAAGA | no amp. |
| Gverm_7244 | KT232124 | (AAGG)_20_ | F: CTGCATCAACACGATTACGC  R: GATATGGGTGGACGAGTGCT | no amp. |
| Gverm_8378 | KT232125 | (AC)_20_ | F: CCAACCTCTCCTCCTGTTTG  R: TGTACGCTGCAATGCTGAAC | no amp. |
| Gverm_8448 | KT232126 | (ACC)_8_ | F: AACTGGAACTGCAAACAATGG  R: GCTTGTTGATGAGCCTGTTG | no amp. |
| Gverm_8535 | KT232127 | (AC)_11_ | F: CGTACAGAATGGGGGATTTG  R: GGAAGATGGATGTGCAGGTT | no amp. |
| Gverm_11521 | KT232128 | (AC)_11_ | F: AAAGGTGGCATTGAGTCAGC  R: TTCATTTGCCACTCTCTAAGCA | no amp. |
| Gverm_11615 | KT232129 | (ATCC)_17_ | F: TGAAACGCCAATAAGTTTCTGTT  R: CACTGTTAGACAACCCAGAAAGA | no amp. |
| Gverm_11818 | KT232130 | (AAGT)_18_ | F: AAGGAAGAAGCATCACCTTCA  R: TCGATTCTGATTGGTTAATAGCAA | no amp. |
