# Supplementary material for: Development and characterization of microsatellite loci for the haploid–diploid red seaweed Gracilaria vermiculophylla
Source: PeerJ. 2015 Aug 11;3:e1159. doi: 10.7717/peerj.1159 (PMC4558075; doi:10.7717/peerj.1159)
Supplement: Table S2 — Frequencies were directly estimated in the haploid subpopulations, whereas frequencies in each of the diploid subpopulations at Akkeshi, Elkhorn Slough, Fort Johnson and Nordstrand were calculated using maximum likelihood and the software MLNullFreq (Kalinowski & Taper, 2006). [file peerj-03-1159-s003.docx]

Table S2. Null allele frequencies for the microsatellite loci developed for *Gracilaria vermiculophylla*. Frequencies were directly estimated in the haploid subpopulations, whereas frequencies in each of the diploid subpopulations at Akkeshi, Elkhorn Slough, Fort Johnson and Nordstrand were calculated using maximum likelihood and the software MLNullFreq (Kalinowski & Taper 2006).

| **Locus** | **Elkhorn** | |  | **Akkeshi** | **Fort Johnson** | **Nordstrand** |
| --- | --- | --- | --- | --- | --- | --- |
|  | **Haploids** | **Diploids** |  | **Diploids** | **Diploids** | **Diploids** |
| Gverm_5276 | 0 | 0.185 |  | 0 | 0 | 0 |
| Gverm_6311 | 0 | 0.161 |  | 0 | 0 | 0 |
| Gverm_8036 | 0 | 0.115 |  | 0 | 0 | 0 |
| Gverm_3003 | 0 | 0.125 |  | 0 | 0 | 0 |
| Gverm_1203 | 0 | 0 |  | 0 | 0 | 0 |
| Gverm_1803 | 0.019 | 0.207 |  | 0.115 | 0 | 0 |
| Gverm_804 | 0 | 0 |  | 0 | 0 | 0 |
| Gverm_10367 | 0 | 0 |  | 0 | 0 | 0 |
| Gverm_2790 | 0.019 | 0 |  | 0 | 0 | 0 |
