# Supplementary material for: Development and characterization of microsatellite loci for the haploid–diploid red seaweed Gracilaria vermiculophylla
Source: PeerJ. 2015 Aug 11;3:e1159. doi: 10.7717/peerj.1159 (PMC4558075; doi:10.7717/peerj.1159)
Supplement: Table S3 — This includes number of pooled size classes used in regression analysis (following Wattier et al., 1998), No. of classes, and linear regression statistics. Loci Gverm_10367 and Gverm_2790 only exhibited two alleles in our sampled populations and consequently, short allele dominance analysis was not applicable (NA). [file peerj-03-1159-s004.docx]

Table S3. Short allele dominance analysis for microsatellite loci developed for *Gracilaria vermiculophylla* including number of pooled size classes used in regression analysis (following Wattier *et al.* 1998), *No. of classes*, and linear regression statistics. Loci Gverm_10367 and Gverm_2790 only exhibited two alleles in our sampled populations and consequently, short allele dominance analysis was not applicable (NA).

| **Locus** | **No. of classes** | ***R^2^*** | ***F*** | ***p-value*** |
| --- | --- | --- | --- | --- |
| Gverm_5276 | 4 | -0.443 | 0.0792 | 0.805 |
| Gverm_6311 | 3 | -0.999 | < 0.001 | 0.984 |
| Gverm_8036 | 3 | 0.294 | 1.83 | 0.405 |
| Gverm_3003 | 3 | -0.805 | 0.108 | 0.798 |
| Gverm_1203 | 3 | 0.772 | 7.79 | 0.219 |
| Gverm_1803 | 3 | -0.442 | 0.387 | 0.646 |
| Gverm_804 | 4 | -0.265 | 0.371 | 0.604 |
| Gverm_10367 | NA | NA | NA | NA |
| Gverm_2790 | NA | NA | NA | NA |
