# Supplementary material for: Development and characterization of microsatellite loci for the haploid–diploid red seaweed Gracilaria vermiculophylla
Source: PeerJ. 2015 Aug 11;3:e1159. doi: 10.7717/peerj.1159 (PMC4558075; doi:10.7717/peerj.1159)
Supplement: Table S4 — Darkened cells indicate pairs of loci that show significant linkage disequilibrium after Bonferroni correction (p-value threshold < 0.006 at α = 0.05). [file peerj-03-1159-s005.docx]

Table S4 Linkage disequilibrium analysis for microsatellite loci developed for *Gracilaria vermiculophylla*. Darkened cells indicate pairs of loci that show significant linkage disequilibrium after Bonferroni correction (*p*-value threshold < 0.006 at α = 0.05).

A) Akkeshi

| Gverm_6311 |  |  |  |  |  |  |  |  |
| --- | --- | --- | --- | --- | --- | --- | --- | --- |
| Gverm_8036 |  |  |  |  |  |  |  |  |
| Gverm_3003 |  |  |  |  |  |  |  |  |
| Gverm_1203 |  |  |  |  |  |  |  |  |
| Gverm_1803 |  |  |  |  |  |  |  |  |
| Gverm_804 |  |  |  |  |  |  |  |  |
| Gverm_10367 |  |  |  |  |  |  |  |  |
| Gverm_2790 |  |  |  |  |  |  |  |  |
|  | Gverm_5276 | Gverm_6311 | Gverm_8036 | Gverm_3003 | Gverm_1203 | Gverm_1803 | Gverm_804 | Gverm_10367 |

B) Elkhorn Slough

| Gverm_6311 |  |  |  |  |  |  |  |  |
| --- | --- | --- | --- | --- | --- | --- | --- | --- |
| Gverm_8036 |  |  |  |  |  |  |  |  |
| Gverm_3003 |  |  |  |  |  |  |  |  |
| Gverm_1203 |  |  |  |  |  |  |  |  |
| Gverm_1803 |  |  |  |  |  |  |  |  |
| Gverm_804 |  |  |  |  |  |  |  |  |
| Gverm_10367 |  |  |  |  |  |  |  |  |
| Gverm_2790 |  |  |  |  |  |  |  |  |
|  | Gverm_5276 | Gverm_6311 | Gverm_8036 | Gverm_3003 | Gverm_1203 | Gverm_1803 | Gverm_804 | Gverm_10367 |

C) Fort Johnson

| Gverm_6311 |  |  |  |  |  |  |  |  |
| --- | --- | --- | --- | --- | --- | --- | --- | --- |
| Gverm_8036 |  |  |  |  |  |  |  |  |
| Gverm_3003 |  |  |  |  |  |  |  |  |
| Gverm_1203 |  |  |  |  |  |  |  |  |
| Gverm_1803 |  |  |  |  |  |  |  |  |
| Gverm_804 |  |  |  |  |  |  |  |  |
| Gverm_10367 |  |  |  |  |  |  |  |  |
| Gverm_2790 |  |  |  |  |  |  |  |  |
|  | Gverm_5276 | Gverm_6311 | Gverm_8036 | Gverm_3003 | Gverm_1203 | Gverm_1803 | Gverm_804 | Gverm_10367 |

D) Nordstrand

| Gverm_6311 |  |  |  |  |  |  |  |  |
| --- | --- | --- | --- | --- | --- | --- | --- | --- |
| Gverm_8036 |  |  |  |  |  |  |  |  |
| Gverm_3003 |  |  |  |  |  |  |  |  |
| Gverm_1203 |  |  |  |  |  |  |  |  |
| Gverm_1803 |  |  |  |  |  |  |  |  |
| Gverm_804 |  |  |  |  |  |  |  |  |
| Gverm_10367 |  |  |  |  |  |  |  |  |
| Gverm_2790 |  |  |  |  |  |  |  |  |
|  | Gverm_5276 | Gverm_6311 | Gverm_8036 | Gverm_3003 | Gverm_1203 | Gverm_1803 | Gverm_804 | Gverm_10367 |
