# Supplementary material for: Development and characterization of microsatellite loci for the haploid–diploid red seaweed Gracilaria vermiculophylla
Source: PeerJ. 2015 Aug 11;3:e1159. doi: 10.7717/peerj.1159 (PMC4558075; doi:10.7717/peerj.1159)
Supplement: Table S5 — This includes: number of alleles at each locus, NA, + standard error (SE); mean allelic richness, AE, based on the smallest global sample size of 46 alleles (23 diploid individuals) + SE; mean observed heterozygosity, HO, + SE; mean expected heterozygosity, HE, + SE. [file peerj-03-1159-s006.docx]

Table S5. Genetic features per locus of four populations of *Gracilaria vermiculophylla*, including: number of alleles at each locus, *N_A_*, + standard error (SE); mean allelic richness, *A_E_*, based on the smallest global sample size of 46 alleles (23 diploid individuals) + SE; mean observed heterozygosity, *H_O_*, + SE; mean expected heterozygosity, *H_E_*, + SE.

| **Locus** | **Akkeshi** | | | |  | **Elkhorn Slough** | | | |  | **Fort Johnson** | | | |  | **Nordstrand** | | | |
| --- | --- | --- | --- | --- | --- | --- | --- | --- | --- | --- | --- | --- | --- | --- | --- | --- | --- | --- | --- |
|  | *N_A_* | *A_E_* | *H_O_* | *H_E_* |  | *N_A_* | *A_E_* | *H_O_* | *H_E_* |  | *N_A_* | *A_E_* | *H_o_* | *H_E_* |  | *N_A_* | *A_E_* | *H_O_* | *H_E_* |
| Gverm_5276 | 5 | 4.7 | 0.355 | 0.682 |  | 4 | 3.5 | 0.333 | 0.378 |  | 3 | 3 | 0.632 | 0.524 |  | 3 | 3 | 0.783 | 0.530 |
| Gverm_6311 | 5 | 4.7 | 0.290 | 0.510 |  | 3 | 3 | 0.567 | 0.657 |  | 2 | 2 | 0.526 | 0.417 |  | 2 | 2 | 0.130 | 0.125 |
| Gverm_8036 | 4 | 3.9 | 0.323 | 0.482 |  | 1 | 1 | NA | NA |  | 3 | 3 | 0.947 | 0.659 |  | 2 | 2 | 0.609 | 0.502 |
| Gverm_3003 | 2 | 2 | 0.097 | 0.204 |  | 3 | 3 | 0.500 | 0.447 |  | 2 | 2 | 0.474 | 0.417 |  | 2 | 2 | 0.783 | 0.510 |
| Gverm_1203 | 4 | 3.7 | 0.516 | 0.450 |  | 2 | 2 | 0.600 | 0.499 |  | 2 | 2 | 0.632 | 0.484 |  | 2 | 2 | 0.739 | 0.496 |
| Gverm_1803 | 3 | 3 | 0.258 | 0.593 |  | 3 | 3 | 0.200 | 0.368 |  | 2 | 2 | 0.842 | 0.501 |  | 1 | 1 | NA | NA |
| Gverm_804 | 3 | 2.7 | 0.516 | 0.450 |  | 2 | 2 | 0.600 | 0.499 |  | 2 | 2 | 0.632 | 0.484 |  | 2 | 2 | 0.739 | 0.496 |
| Gverm_10367 | 2 | 1.9 | 0.065 | 0.063 |  | 1 | 1 | NA | NA |  | 1 | 1 | NA | NA |  | 1 | 1 | NA | NA |
| Gverm_2790 | 1 | 1 | NA | NA |  | 1 | 1 | NA | NA |  | 1 | 1 | NA | NA |  | 2 | 2 | 0.957 | 0.510 |
